# Case report on antipsychotic induced catatonia in an incarcerated patient

**DOI:** 10.3389/fpsyt.2023.1092253

**Published:** 2023-08-31

**Authors:** Han Nguyen, Ruslana Cannell, Suman Rollakanti, Johanna Rosenthal, David Seigler

**Affiliations:** ^1^Department of Behavioral Health, Arrowhead Regional Medical Center, Colton, CA, United States; ^2^California University of Science and Medicine, Colton, CA, United States; ^3^Loma Linda University School of Medicine, Loma Linda, CA, United States

**Keywords:** antipsychotic induced catatonia, haloperidol decanoate, extrapyramidal symptoms, first-generation antipsychotics, benzodiazepines, long acting injectable, forensic psychiatry, Bush-Francis scale

## Abstract

Antipsychotic-induced catatonia is an iatrogenic and debilitating adverse reaction, but there is a dearth of recent documented cases. This report describes a 35-year-old incarcerated Korean-American male with a history of unspecified psychosis who presented for antipsychotic induced catatonia after administration of haloperidol decanoate intramuscular (200 mg across the span of 1 week). Neurologic workup was performed including MRI, lumbar puncture, and electroencephalography. Despite an approximate month long hospitalization, benzodiazepine challenge, benztropine trial, and amantadine adjunct, our patient continued to experience bradykinesia, waxy flexibility, and mask-like facies, and was minimally verbally responsive. Several challenges in the treatment of incarcerated individuals at the hospital are highlighted in this case report, including adverse reaction to medication, difficulty of care coordination, and limited access to health records among providers.

## Introduction

Catatonia is a psychomotor syndrome including but not limited to hypokinetic or hyperkinetic movements, stereotyped or negativistic behaviors, and mutism or verbigeration (1). A recent meta-analysis has shown that the mean prevalence of catatonia among subjects diagnosed with a variety of psychiatric or medical conditions is 9.2% (2). Clinically, the Bush-Francis Catatonia Rating Scale (BFCRS) can provide systematic scoring to quantify severity of catatonia (3, 4). The BFCRS is based on 23 examination items; criteria for catatonia is met when two or more of the first 14 items are positive. Higher BFCRS scores indicate either more numerous symptoms or higher severity of symptoms. Mutism, stupor, staring, posturing, and negativism are some of the features of catatonia in this widely used scale (3). Catatonia is most associated with psychiatric conditions, but can also occur as a result of neurologic traumas, autoimmune diseases, metabolic disorders, and medication exposure (e.g., antipsychotics), and more (4). Iatrogenic movement disorders that occur after administration of antipsychotics are termed antipsychotic induced movement disorders, and can include antipsychotic induced catatonia (AIC), parkinsonism, and akathisia.

There have been a limited number of cases of antipsychotic-induced catatonia documented, with the majority reported around the 1980s. The increased use of second-generation over first-generation antipsychotics can explain the decreasing prevalence of antipsychotic-induced catatonia. Of note, “antipsychotic” and “neuroleptic” have historically been used interchangeably to refer to the same class of medications but “antipsychotic” has gained favor as it more accurately describes the ability of these drugs to treat psychoses (5). Second generation antipsychotics are less likely to cause endocrine side effects and extrapyramidal symptoms (EPS) including antipsychotic-induced catatonia compared to first-generation antipsychotics (6, 7). Here, we present a brief summary of the antipsychotic-induced catatonia case reports that have been documented. Following the administration of loxapine, eight individuals had symptoms of catatonia, according to a 1977 report (8). Another case study from 1986 documented a 19-year-old male who developed parkinsonian and catatonic symptoms after accidentally ingesting haloperidol 30 mg by mouth (9). A young female had autonomic disturbances and catatonic symptoms after administration of haloperidol, biperiden, thioridazine, and haloperidol decanoate in a case report (10). In 1992, an adolescent 16 year old male patient treated with trifluoperazine presented to the emergency department with muscle rigidity, muteness, and staring (11). In 2005, psychiatrists in Taiwan documented a case of catatonia after 3 month regimen of zotepine 250 mg/day that progressed to neuroleptic malignant syndrome (NMS) (12). A study by Lee et al. (13) examined 127 cases of catatonia and identified 18 patients that met criteria for neuroleptic induced catatonia; this study independently cited many of the aforementioned studies above (13). Given the low prevalence and the fact that the most recent case report was published over 15 years ago, documenting cases of AIC is necessary to deepen our understanding of this iatrogenic condition.

In this case report, we focus on haloperidol. Patients are often prescribed haloperidol ranging from 1 to 40 mg/day (14). Haloperidol can be administered as the long acting injectable haloperidol decanoate to overcome medication nonadherence. The loading dose for haloperidol decanoate is 10–20 times the oral dose to achieve steady state (15). Inadequate loading can result in sub-therapeutic levels in the plasma or prolonged course of treatment. A reasonable and even routine dosing regimen would be haloperidol decanoate 100 mg intramuscular (IM), followed by haloperidol decanoate 100 mg IM on week 2, and with coadministration of haloperidol oral overlap. Another loading strategy can be haloperidol decanoate 300 mg IM every 1–2 weeks for two doses, then measuring plasma levels to determine further added dosings (14).

This case report also highlights several aspects of the challenges faced in the care of incarcerated individuals, and the need for improved care coordination (16). It is well known that the prevalence of incarcerated individuals requiring mental health care is remarkably high. After the deinstitutionalization of mental illness in the 1950s, some experts used the term “transinstitutionalization” to describe the large numbers of individuals with psychiatric illness who become incarcerated (15). A systematic review of 22,790 prisoners across 12 countries has estimated that among incarcerated men, about 3.7% had psychotic disorder, 10% had major depression, and up to 65% had a personality disorder. Among incarcerated women, the prevalence of these conditions was 4, 12, and 42%, respectively (17). Given the high prevalence of psychiatric disorders among the prison patient population, the American Psychiatric Association has recommended that incarcerated individuals should be provided with the same level of mental health treatment that is available in the general community (18). Improvements in care coordination can be made to prevent adverse outcomes in our incarcerated patients (19, 20).

## Case presentation

A 35-year-old incarcerated Korean-American male with a history of unspecified psychosis presented to our hospital for extrapyramidal symptoms (EPS) after administration of haloperidol decanoate IM.

The patient was incarcerated at a jail site outside of our county where he received haloperidol decanoate 100 mg IM. On that same day, he was transferred to our county’s detention center to determine competency to stand trial. Here, he was started on a course of risperidone 6 mg PO daily for a total of 10 days. The patient was in treatment with both haloperidol and risperidone in addition to valproic acid while in jail. Due to limited access to medical records, it is unclear to these authors the reason why the patient was a candidate for dual antipsychotic therapy. Ten days after the initial haloperidol decanoate dose, the patient received one additional dose of haloperidol decanoate 100 mg IM on. Full psychiatric history was limited in this study due to the patient’s incarceration at two jail sites, limited access to records, limited access to collateral information, and the patient who was only minimally interactive with the environment. There was significant difficulty in obtaining the patient’s medical records; to glean more information, the investigators of this case report called the jail, eventually reaching the charge nurse, who then read the notes of the jail psychiatrist.

**Figure 1:**
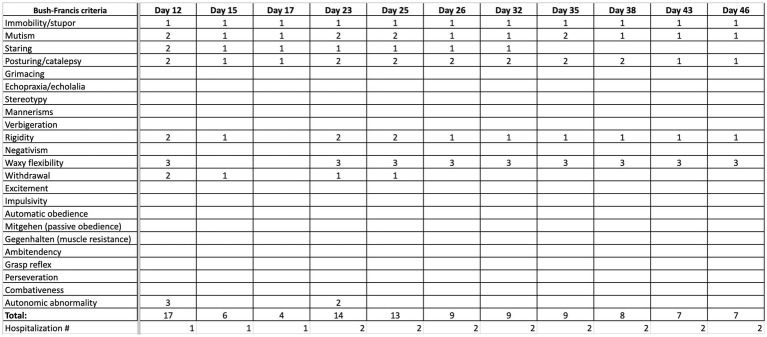
Summary of progression of clinical status, quantified by Bush Francis scoring system (BFCRS) for catatonia.

**Figure 2:**
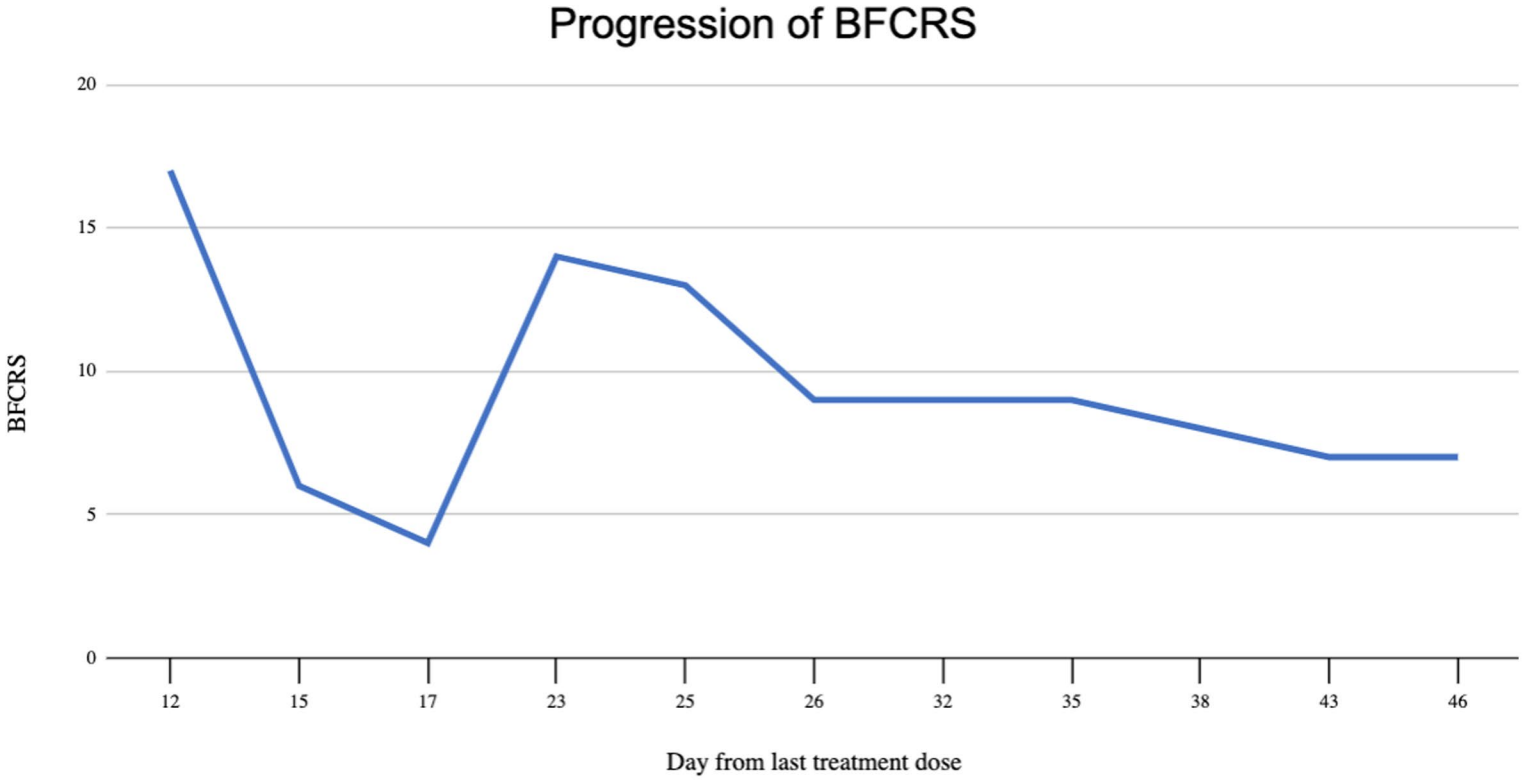
Graphical depiction of Bush Francis catatonia rating scale score.

The day following the second IM dose, he was admitted to our hospital for chief complaint of “EPS after haloperidol IM.” His symptoms included bradykinesia, stooped posture, mask-like facies, bradykinesia, diaphoresis, drooling, and suicidal ideation. The patient scored 17 points on the BFCRS. A summary and detailed scoring of the patient’s clinical status in terms of BFCRS is depicted in Figures 1, 2. On arrival, his vital signs were significant for tachycardia and hypertension: temperature 97.1 F (36.2 C), pulse 115 beats/min, respiratory rate 18 breaths/min, and blood pressure 158/109 mmHg. His initial laboratory findings were significant for elevated creatine kinase 1,335 U/L, increased anion gap of 20, and total bilirubin of 1.3 mg/dL, AST slightly elevated (57 IU/L) but ALT was within normal limits (35 IU/L). There was no leukocytosis (WBC 6.0 TH/UL), no thromobocytopenia (platelets 194 TH/UL). Iron panel, and anti-NMDA receptor IgG tests were not obtained. Patient also presented with active suicidal ideation stating he was at the hospital “to kill myself.” The Poison Control Center was contacted due to possible supratherapeutic dosage of haloperidol, and recommended IV fluid hydration with intramuscular diphenhydramine. Poison Control also confirmed the low concern for NMS given lack of hyperthermia, and lead pipe rigidity. The anion gap was thought to be secondary to lactic acidosis in the context of elevated creatine kinase in addition to poor oral intake. Electrocardiogram demonstrated sinus tachycardia with QTc of 403 ms. Of note, the patient was found to have a lice infestation of the scalp and was treated with permethrin 5% cream during hospitalization.

**Figure 3:**
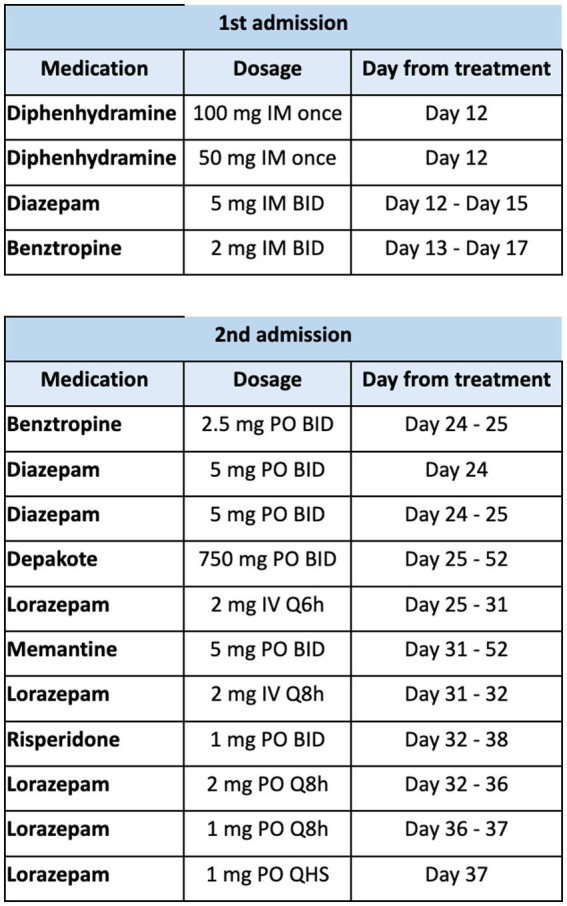
Summary of medication therapy throughout hospitalization at our facility.

In terms of treatment, antipsychotic therapy was immediately held. Medication therapy included two consecutive doses of diphenhydramine 50 mg IM and a total of 2 L of Lactated Ringer’s solution in the emergency department. The patient was continued on a 3-day course of diazepam 5 mg IM BID, maintenance fluids (dextrose 5% in 0.45% normal saline with 20 meq of potassium infusing at 70 mL/h for 4 days), and a 5 day course of benztropine 2 mg PO BID. A summary of the medication therapy course is depicted in Figure 3. After day 2 of admission, the patient became alert and oriented fully to self, place, time, and situation. Vital signs normalized, the anion gap resolved, and his suicidal ideation eventually resolved by end of admission. We continued to hold antipsychotic therapy given his elevated BFCRS score, and continued benztropine 2 mg PO BID. The patient had improvement of his motor symptoms to BFCRS of 4 and he was medically cleared. He was discharged on the sixth day of hospitalization and recommended to continue benztropine 2 mg PO BID and suicide watch in jail.

Within the next week, the patient returned to our emergency department from the detention center for another episode of “EPS,” with similar symptoms that included rigidity, increased muscle tone, and resting tremor. BFCRS score was 14. Reportedly, antipsychotics were not resumed in the interim. One provider noted “over the last month he has felt like it has been very hard to move and he feels very stiff.” On arrival, his vital signs again were significant for tachycardia and hypertension: temperature 97.9 F (36.6 C), pulse 104 beats/min, respiratory rate 16 breaths/min, blood pressure 151/98 mmHg, and oxygen saturation at 96% on room air. Laboratory studies showed elevated creatine kinase 685 U/L, and identical anion gap of 20. There was no leukocytosis, WBC 4.8, platelets within normal limits at 290 TH/UL, Electrocardiogram demonstrated sinus tachycardia with QTc 366 ms.

The patient was treated with diphenhydramine 50 mg IM as well as a 2-day course of benztropine 2.5 mg BID PO and diazepam 5 mg BID PO. Patient was admitted, and Psychiatry Consult and Liaison service was on board. After 2 days of minimal improvement, the patient’s benzodiazepine regimen was changed to lorazepam 2 mg IV q6, which was gradually tapered across 12 days. Patient received memantine 5 mg PO BID for the rest of this admission (27 days), which was on formulary and was added for the intention of reducing antipsychotic induced EPS. Patient was also re-started on home medication of valproic acid 750 mg PO BID, which he continued until day of discharge. Patient demonstrated significant clinical improvement per documentation, and he was seen attempting to eat his breakfast after lorazepam was started. The Neurology team was consulted for persistence of drug induced parkinsonism, and for consideration of a dopamine agonist. Neurology recommended further workup including brain magnetic resonance imaging (MRI), electroencephalography (EEG), and lumbar puncture to rule out other causes. Haloperidol serum levels were obtained about 2 months after administration of the second dose of haloperidol decanoate, which resulted 1 ng/mL (subtherapeutic). While awaiting testing, the patient demonstrated self-injurious behaviors after he wrapped his bedsheets around his neck to strangle himself. When asked about his behaviors, he was unable to elaborate on any particular reason or cause. He was not observed to be responding to internal stimuli during the hospitalization, but given his psychiatric history and mutism we suspected worsening of psychosis. Subsequently, patient resumed risperidone 1 mg po bid for 6 days until it was discontinued secondary to minimal improvement of EPS symptoms and resolution of self-harming behaviors with the absence of suicidal ideation.

Neurological workup was performed due to persistence of movement disorder symptoms. Brain MRI demonstrated no acute abnormalities. EEG was performed which was read as abnormal due to generalized slowing of the predominant rhythms as well as frequent episodes in which the patient had bursts of bifrontal slow activity or parietal spikes. Lumbar puncture performed with unremarkable cerebral spinal fluid studies; neurosyphilis, West Nile virus was ruled out. Additionally, serum studies ruled out viral encephalitis (adenovirus, cytomegalovirus, herpes simplex virus, 1 + 2, enterovirus, varicella zoster virus, and human immunodeficiency virus). A dopamine agonist such as pramiprexole was considered, but the risk of worsening psychosis and suicidal ideation outweighed the potential benefit in this patient who had antipsychotic induced parkinsonism vs. less likely primary parkinsonism. After completion of the above investigations, the Neurology team suggested the patient’s symptoms were secondary to antipsychotic induced EPS, and less likely anatomical or infectious in origin.

Throughout his hospital stay, particularly after 15 days of scheduled benzodiazepines, the patient demonstrated improvement in autonomic abnormalities, mutism, catalepsy, and ability to complete activities of daily living. However, he remained bradykinetic, and continued to have waxy flexibility and mask-like facies. His BFCRS upon discharge remained at 7. The patient continued to have an elevated BFCRS score, but his psychiatric and neurologic status were determined to be improving and stable for outpatient management. The patient was ultimately discharged on treatment of valproic acid 500 mg PO BID.

### Investigations and limitations

#### EEG

Technical aspect: This EEG was recorded utilizing the International 10–20 system with 21 recording electrodes and digital and video EEG monitoring.

Description of the Recording: With the eyes closed the predominant rhythm was a poorly modulated disorganized mixture of 6 and 7 cps activity that was generalized. It was 35–40 μV.

Duration of study: 20 min.

## Results

Early in the recording the patient set up and rubbed his head leading to a great deal of electrode artifact but may have actually been some abnormalities under the artifact the patient had another episode of rhythmic activity when he was touching the electrodes several times later in the recording when he was resting quietly rhythmic generalized abnormalities were seen lasting for 3–5 s.

### Interpretation

This EEG is abnormal due to generalized slowing of the predominant rhythms as well as frequent episodes in which the patient had bursts of bifrontal slow activity or parietal spikes. The patient was somewhat uncooperative and there is movement artifact some of the episodes may have simply been electrode artifact related to patient positioning.

### MRI brain

Findings: There is mild motion artifact. The brain parenchyma demonstrates preserved gray-white differentiation. There is mild atrophy. No midline shift. Basal cistern is patent. There is no acute diffusion restriction or gradient sequence abnormality. No abnormal enhancement is seen. The pituitary gland and brainstem are within normal limit.

Impression: No acute intracranial abnormality.

**Figure 4:**
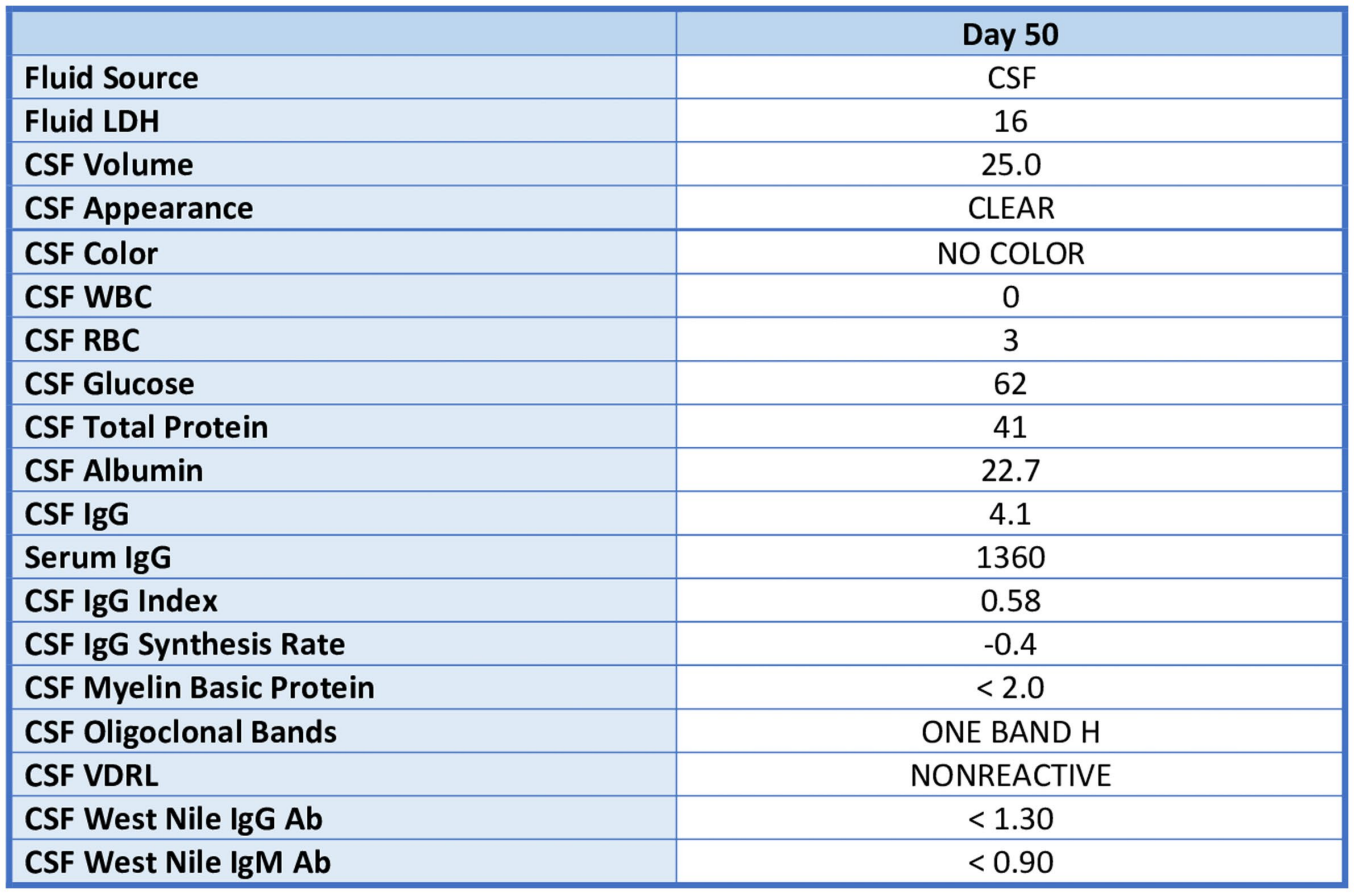
Lumbar puncture results.

Lumbar puncture results unremarkable; see Figure 4.

Viral cultures lab results collected 11/1/21:

Negative for adenovirus, CMV, HSV 1 and 2, enterovirus, and VZV.

Haloperidol serum level collected:

1 (reference range 5–15 ng/mL).

Haloperidol level report: subtherapeutic.

There are several limitations to our case report. In addition to limited history, several studies were unavailable at our county facility. A DaTscan could have allowed us to differentiate organic vs. drug induced parkinsonism. We would expect this patient to have normal dopamine uptake at the striatum with a DaTscan (which would be consistent with drug-induced parkinsonism) as opposed to reduced striatal dopamine uptake (consistent with neurodegenerative cause of parkinsonism). Given that we were part of consult services, BFCRS measurements were estimates based on physical exam findings and surveying of examiners. In terms of treatment, our facility did not have amantadine on formulary and memantine was used instead, as it has been shown to reduce EPS symptoms such as bradykinesia (21). Amantadine has been identified as a reasonable treatment for drug-induced parkinsonism (22). Lastly, while this patient would have been a candidate for electroconvulsive therapy for his catatonic presentation, our facility does not currently provide this service.

## Discussion

Documented cases of AIC are sparse, and the most recent case in our literature review was over 15 years ago. This could be due to low prevalence, as well as the transition toward using second generation antipsychotics over first generation antipsychotics in clinical psychiatric practice. The theoretical benefit of overall lower dopamine D2 receptor affinity in second generation antipsychotics is supported by the decreased prevalence of AIC.

The patient received a total of haloperidol decanoate 200 mg IM within 10 days with risperidone 6 mg po daily x 10 days. Although these are moderate-high doses of two high potency antipsychotics, they are often used as monotherapy for patients with psychotic spectrum disorders, especially in the forensic setting. Thus, it is reasonable to explore other contributing factors to the patient’s presentation. Contributing to his presentation could be the use of dual antipsychotic therapy (risperidone and haloperidol). Since we have limited prior history, it is difficult to determine why he required dual antipsychotics. Furthermore, given the dose of risperidone 6 mg daily would reach 80% D2 receptor binding affinity, this would be comparable to a first generation antipsychotic (23). In other words, the patient effectively was receiving two high D2 affinity antipsychotics. It is also important to note that the route of administration was incredibly important in this case. Since the patient received two doses of haloperidol decanoate long acting injectable, the duration of adverse symptoms was most likely longer than if he had only received oral medication.

The patient came to us from another county’s jail with an unclear psychiatric history, limited information about his prior presentation, and a diagnosis of “unspecified psychosis.” Due to jail policies, we were not permitted to call the patient’s family to obtain more information about his history. In light of a questionable psychiatric history, obtaining this information would have given us a deeper understanding of his presenting condition. Additionally, the patient himself was a poor historian and provided very little history. One differential diagnosis considered was malingering with possible secondary gain being a prolonged hospital stay. However, he presented with autonomic instability and had cogwheel rigidity on exam, both symptoms of which would be difficult to willfully produce. Thus, we had lower suspicion of the patient feigning symptoms. Despite limited history, a unique feature of this case presentation is the extensive neurologic workup performed (MRI, EEG, lumbar puncture, and repeated clinical exam by neurologist), allowing us to rule out many other neurologic differential diagnoses. In terms of his symptoms, differential diagnoses include AIC, antipsychotic induced parkinsonism, or early NMS.

Initially, the treatment teams were worried that the patient may have been a slow metabolizer of medication, especially given his East Asian ethnicity. However, the possibility that patient was a slow metabolizer of medication was ruled out with the haloperidol serum level demonstrating low serum levels, and not supratherapeutic. This likely is attributed to both administrations of the long acting injectable haloperidol decanoate.

Several challenges in the treatment of incarcerated individuals are highlighted in this case report, including patient decompensation (worsening psychosis and presence of lice), poor care coordination, and limited access to health records among providers. The prevalence of mental illness in incarcerated individuals is incredibly high, and the majority often require coordination to higher levels of care while in jail (16). Despite being one of the largest providers of mental health services, access to treatment can be limited due to many factors, some of which include lack of resources, overrepresentation of individuals with mental disorders, and expenditure (24). Our patient in this report presented with a significant adverse event after two high potency antipsychotics, in addition to having a lice infestation. Furthermore, it was incredibly difficult to obtain his medical history. Records were not easily accessible, and an unreasonable amount of time and resources were used to obtain basic medical information between two community providers. The difficulty of coordinating care in this case could have been due to the complexity of the task, considering patient interest, efficiency at the state and facility level, budgetary needs, leadership, and limited resources. Easing access to medical data would have been imperative in the care of this patient (20). Improving coordination can better continuity of care between facilities in this vulnerable population of patients (20).

## Data availability statement

The original contributions presented in the study are included in the article/supplementary material, further inquiries can be directed to the corresponding author.

## Ethics statement

Written informed consent was obtained from the individual(s) for the publication of any potentially identifiable images or data included in this article.

## Author contributions

HN and DS conceived of the presented idea. HN wrote the manuscript with support from RC. SR, DS, and JR supervised the project. HN, RC, SR, JR, and DS discussed the findings. All authors contributed to the article and approved the submitted version.

## Conflict of interest

The authors declare that the research was conducted in the absence of any commercial or financial relationships that could be construed as a potential conflict of interest.

## Publisher’s note

All claims expressed in this article are solely those of the authors and do not necessarily represent those of their affiliated organizations, or those of the publisher, the editors and the reviewers. Any product that may be evaluated in this article, or claim that may be made by its manufacturer, is not guaranteed or endorsed by the publisher.
